# Down-regulation of biofilm-associated genes in *mecA*-positive methicillin-resistant *S. aureus* treated with *M. communis* extract and its antibacterial activity

**DOI:** 10.1186/s13568-021-01247-z

**Published:** 2021-06-10

**Authors:** Moj Khaleghi, Sadegh Khorrami

**Affiliations:** 1 Department of Biology, Faculty of Sciences, Shahid Bahonar University of Kerman, Kerman, Iran; 2 Research and Technology Institute of Plant Production, Shahid Bahonar University of Kerman, Kerman, Iran

**Keywords:** *M. communis*, methicillin-resistant *S. aureus*, antibacterial agent, antibiofilm agent, biofilm-associated genes

## Abstract

Considering the prevalence of resistance to antibiotics, the discovery of effective agents against resistant pathogens is of extreme urgency. Herein, 26 *mecA*-positive methicillin-resistant *S. aureus* (MRSA) isolated from clinical samples were identified, and their resistance to 11 antibiotics was investigated. Next, the antibacterial and anti-biofilm activity of the ethanolic extract of *M. communis* on these strains was evaluated. Furthermore, the effect of this extract on the expression of biofilm-associated genes, *icaA*, *icaD*, *bap*, *sarA*, and *agr*, was studied. According to the results, all isolated strains were multidrug-resistant and showed resistance to oxacillin and tetracycline. Also, 96.15 and 88.46 % of them were resistant to gentamicin and erythromycin. However, the extract could effectively combat the strains. The minimum inhibitory concentration (MIC) against different strains ranged from 1.56 to 25 mg/ml and the minimum bactericidal concentration (MBC) was between 3.125 and 50 mg/ml. Even though most MRSA (67 %) strongly produced biofilm, the sub-MIC concentration of the extract destroyed the pre-formed biofilm and affected the bacterial cells inside the biofilm. It could also inhibit biofilm development by significantly decreasing the expression of *icaA*, *icaD*, *sarA* and *bap* genes involved in biofilm formation and development. In conclusion, the extract inhibits biofilm formation, ruins pre-formed biofilm, and kills cells living inside the biofilm. Furthermore, it down-regulates the expression of necessary genes and nips the biofilm formation in the bud.

## Introduction

As one of the leading causes of nosocomial infections, the treatment of *Staphylococcus aureus* infections are arduous due to the emergence of antibiotic-resistant strains. Methicillin-resistant *S. aureus* (MRSA), particularly, is resistant to penicillin and other semi-synthetic beta-lactams, such as methicillin and oxacillin (Klein et al. 2007). The first outbreak of MRSA in European hospitals occurred in the 1960 s. During the 1970 s *S. aureus* strains established resistance to several antibiotics and came to the cause of many infections in American and British hospitals. In the 1980 s, MRSA was reported as a major caue of nosocomial infections (Akinyemi et al. 2005).

What makes the *S. aureus* a virulent strain are both its adhesion and invasion properties. The adhesion ability is associated with biofilm formation and results in a sheltered life toward antibiotics. A microbial biofilm consists of a community of microbial cells that irreversibly attach to the substrate or each other. This community produces a surrounding matrix of extracellular polymeric materials (Parastan et al. 2020). Intercellular adhesion polysaccharide (PIA), which is encoded by the intercellular adhesion (ica) locus, specifically the *icaA* gene, is composed of a linear 1,6-linked glycosaminoglycan and made from UDP-N-acetylglucosamine by N-acetylglucosaminyltransferase. The cooperation of *icaA* and *icaD* genes leads to phenotypic expression of capsular polysaccharides (Fariba et al. 2020; Melo et al. 2013). In addition, the *ica*-independent mechanism plays a vital role in the formation of bacterial biofilms. For example, *bap*, which encodes Bap surface protein, aids intracellular binding and biofilm formation. Moreover, it has been determined that suppression of the *agr* quorum-sensing system is necessary for biofilm formation. This system stimulates biofilm detachment by increasing auto-inducing peptides (AIPS) or decreasing glucose (Marques et al. 2017). In this context, *sarA* locus encodes a DNA-binding protein (SarA) that is required for maximal expression of *agr* and RNAIII promoters under different growth conditions.

Combating the cells living in a biofilm usually needs much higher antibiotic concentrations during a prolonged period, and the current strategies often fail, resulting in infection persistence. In addition to therapeutic limitations, when the bacteria grow in medical devices, their biofilms can be the origin of infections. The difficulty imposed by biofilm has mobilized researchers worldwide to prospect or develop innovative solutions to control biofilm (Dzianach et al. 2019; Ribeiro et al. 2016). In this context, valid evidence demonstrated that plant products, as a precious source of bioactive compounds with antimicrobial and chemo-preventive properties, can be applied to overcome the biofilm-related challenges (Makvandi et al. 2019; Wang et al. 2020). In the last decades, novel approaches have been rapidly developed to inhibit biofilm formation using plant products, and promising results have been achieved.

Myrtle (*Myrtus communis* L.) is a medicinal herb endemic of the Middle East area and grows spontaneously in Iran, Spain, France, Greece, Turkey, Algeria, Morocco, Croatia, and Montenegro (Aleksic and Knezevic 2014). It is a very aromatic plant because its leaf, flower, and fruit glands are rich in essential oil (Aleksic and Knezevic, 2014), and the indigenous people make use of its culinary and medicinal properties since antiquity for the treatment of lung disorders. Furthermore, the antiseptic, anti-inflammatory, mucolytic, carminative, and astringent properties of this plant have been reported. Recently, it has been revealed that *M. communis* extracts possess antioxidant, analgesic, antibacterial, antifungal, and antibiofilm activities (Asgarpanah and Ariamanesh 2015; Zadeh et al. 2020). The study recently published by our group determined the chemical compounds of the extract via GC/MS analyses (Zadeh et al. 2020). As represented in Table 1, the Myrtle extract composes of six main organic compounds. Among them, 2-Furancarboxaldehyde,5-(hydroxymethyl), and 7-Isopropyl-7-methyl-nona-3,5-diene-2,8-dione, with about 42 and 13 % of the total, are the most dominants, respectively.

**Table 1:** The major constituents of the extract of *M. communis* ethanolic extract (Zadeh et al. 2020)

No.	Retention time(min)	Molecular formula	Compound name	% of total
1	13.169	C_6_H_8_O_4_	4 H-Pyran-4-one, 2,3-dihydro-3,5-dihydroxy-6-methyl	5.474
2	17.566	C_6_H_6_O_3_	2-Furancarboxaldehyde, 5-(hydroxymethyl)	42.396
3	21.531	C_13_H_20_O_2_	2,10,10-Trimethyl-6-methylene-1-oxaspiro[4.5]decan-7-one	1.284
4	28.234	C_13_H_20_O_2_	7-Isopropyl-7-methyl-nona-3,5-diene-2,8-dione	12.764
5	35.527	C_22_H_42_O	Z-5-Methyl-6-heneicosen-11-one	4.230
6	36.482	C_14_H_28_	3-Heptene, 2,2,3,5,5,6,6-heptamethyl	5.360

Having said that, studies addressing the detailed molecular mechanisms involved in the biological activities of this extract are still limited. In this study, the antibacterial and anti-biofilm effect of the *M. communis* extract on methicillin-resistant *S. aureus* isolated from clinical samples was investigated and the impact of this plant extract on the expression of *icaA*, *icaD*, *bap*, *sarA*, and *agr* genes was studied.

## Materials and methods

### Isolation of strains

The *S. aureus* strains used in this study had been initially isolated from the wound, abscess, ear, urine, blood, and bronchoalveolar lavage samples collected during one year by the microbiology laboratory of Afzalipour and Shafa hospitals of Kerman province, Iran. The samples were cultured on the Blood Agar medium (Merck, Germany) and incubated at 37 °C for 24 h. After Gram staining, the gram-positive cocci strains were chosen for biochemical tests, including catalase, coagulase, mannitol fermentation, and oxidase, as previously described in the literature (Jafari-Nasab et al. 2021). The identified isolates were stored in a medium containing 20 % glycerol at − 20 °C to be used in further tests.

### Identification of methicillin-resistant *S. aureus* strains and their antibiotic resistance

The methicillin resistance of 40 *S. aureus* isolates were tested by using the oxacillin agar screen method on the Muller Hinton agar modified by adding NaCl (4 %) + oxacillin (6 µg/ml) (Dhanalakshmi et al. 2012). The strains that could grow on this medium were then tested to be revealed whether they carry the *mecA* gene (Kot et al. 2020; Ubukata et al. 1989), as described in the next section.

The disc diffusion method was conducted to determine the antibiotic resistance pattern of the identified methicillin-resistant *S. aureus* strains to Vancomycin (30 µg), Ciprofloxacin (5 µg), Erythromycin (15 µg), Tetracycline (30 µg), Gentamycin (10 µg), Ceftriaxone (30 µg), Amikacin (30 µg), Mupirocin (5 µg), Oxacillin (30 µg), Chloramphenicol (30 µg) and Trimethoprim/Sulfamethoxazole (1.25/23.75 µg) purchased from MAST Group, Merseyside, UK.

### Screening the *mecA* and genes encoding adhesion factors and biofilm

The extraction of DNA from these isolates was performed using enzymatic digestion of lysozyme (Araújo et al. 2004). Primers shown in Table 2 were used to check the presence of the *mecA* gene, as an MRSA indicator (Kot et al. 2018), as well as *icaA*, *icaD*, *bap* and *sarA* genes, as biofilm-associated genes (O’Gara 2007), in each of the strains. The PCR reaction consisted of 1 µl of template DNA, 2.5 µl of Buffer PCR 1x, 200µM dNTP and 0.4 µ rivers and forward primers and 1 unit of Taq DNA Polymerase enzyme. The total reaction volume was 25 µl. The amplification cycles were carried out in a thermal cycler (Mastercycler; Eppendorf). The reaction condition was optimized to be 94 °C for 4 min, as initial denaturation, followed by 35 cycles of 94 °C for 60 s, annealing for 60 s and 72 °C for 60 s, and a final extension step at 72 °C for 10 min. Finally, PCR products were analyzed by agarose gel electrophoresis (1 %).

**Table 2:** The primers were used to screen the presence of the *mecA* and genes encoding adhesion factors and biofilm

genes	Primer sequence PCR	product size (bp)	References
*mec*A	5’-ATCGATGGTAAAGGTTGG-3’ 5’-AGTTCTGCAGTACCGGATTTG-3’	533	(Al-Ali et al. 2014)
*icaA*	5’- TGGCTGTATTAAGCGAAGTC − 3’ 5’- CCTCTGTCTGGGCTTGACC − 3’	669	(Martins et al. 2015)
*icaD*	5’-AAACGTAAGAGAGGTGG-3’ 5’-GGCAATATGATCAAGATAC-3’	381	(Vasudevan et al. 2003)
*bap*〹	5’-CCC TAT ATC GAA GGT GTA GAA TTG-3’ 5’-GCTGTTGAAGTTAATACTGTACCTGC-3’	971	(Cucarella et al. 2004)
*sar*A	5’-TTAGCTTTGAAGAATTCGCTGT-3’ 5’-TTCAATTTCGTTGTTTGCTTC-3’	275	(Padmapriya et al. 2003)
*agr*〹	5’-GTAGAGCCGTATTGATTCC-3’ 5’-GTATTTCATCTCTTTAAGG-3’	463	(Moore and Lindsay, 2001)
*16 S rRNA*〹	5’-GTA GGT GGC AAG CGT TAT CC-3’ 5’-CGC ACA TCA GCG TCA G-3’	228	(Moore and Lindsay, 2001)

To narrow down the number of examined strains, they were firstly tested for their sensitivity toward the *M. communis* extract; then the most susceptible ones were chosen for more detailed studies.

### Preparation of *M. communis* extract

The myrtle (*M. communis*) plant was collected from Mahan city, Kerman province, Iran, in early spring. After identification by the Herbarium of the Department of Biology, Shahid Bahonar University of Kerman (Herbarium Code: KF 1356), the plant’s leaves were dried in the shade and powdered. Then, following the method described by Amensour et al. the extraction was performed (Amensour et al. 2010). Briefly, 100 g of the powdered plant leaves were immersed in 1 L of ethanol and shaken at 25 °C for 48 h. After this time, the samples were filtered by filter paper Wattman No. 4, and the filtrate was concentrated by a rotary evaporator at 40 °C and dried at room temperature. The dried extract was then stored at − 20 °C. To supply intended concentrations, the extract was dissolved in dimethylsulfoxide (DMSO) before each test.

### Evaluation of the extract antibacterial properties

The antibacterial activity of the extract against the isolated MRSA was evaluated by the well-diffusion method (Khorrami et al. 2020). First, the wells with 6 mm diameter were embedded on Muller-Hinton agar medium. Next, the overnight MRSA strains were cultured on the media after standardization to 0.5 McFarland standard. Then, 50 µl of the extract (50 mg/ml) was poured into the wells. After that, the plates were incubated at 37 °C for 24 h, followed by measuring the diameter of the growth inhibition zone (mm). In this test, the *S. aureus* ATCC 33,591 purchased from Pasteur Institute, Tehran, Iran was used as a standard strain.

### MIC and MBC determination

The minimum inhibitory and bactericidal concentrations (MIC and MBC, respectively) of the ethanolic extract were determined according to the Clinical and Laboratory Standards Institute (CLSI) (Cockerill 2011). Briefly, 150 µl of the MHB medium inoculated with the overnight bacterial suspension and adjusted to 0.5 McFarland were poured into the wells of a 96-well plate, then 50 µl of each extract concentration (from 0.05 to 50 mg/ml) was added to the wells and the plate was incubated for 24 h. After this time, the lowest concentration at which no bacterial growth was observed was considered MIC. Furthermore, 10 µl of the cultures were transferred to an extract-free MHA medium and incubated to determine the MBC. The MBC is defined as the lowest concentration at which no bacterial cells grow on an extract-free medium (Khorrami et al. 2019, 2018).

### Biofilm formation assessment

The microtiter plate method was used to evaluate the biofilm formation and adhesion strength of the opted strains (Khaleghi et al. 2019; Zangeneh et al. 2020). Toward this end, an overnight culture of each strain was first prepared in TSB medium (Merck, Germany) and adjusted to 0.5 McFarland standard. Next, 200 µl of the microbial suspension was added to the wells of a 96-well microplate and incubated for 24 h at 37 °C, without movement. The wells were then emptied and the biofilm structure was fixed using ethanol (96 %) and stained with crystal violet (2 %). After washing the surplus paint, 150 µl acid acetic (33 %) was poured into wells, and the absorbance of each well was measured with an ELISA reader (Biotek-EI800) at 492 nm. Finally, the data were prescribed as previously described (Jafari-Nasab et al. 2021). The strains were stored at − 20 °C to use in further tests.

### Anti-biofilm activity of the extract

To evaluate the ability of *M. communis* extract to inhibit biofilm formation, as well as to determine the minimum biofilm-inhibitory concentration (MBIC), the microtiter plate method was applied according to the method previously described by Khaleghi et al. (Khaleghi et al. 2019). In this test, the biofilm inhibitory properties of different concentrations of the extract (0.05–50 mg/ml) were investigated by adding 50 µl of the extract solution into the 100 µl of bacterial suspensions and incubating for 24 h. The percentage of inhibition was calculated according to Eq. 1. An uncultured medium was used as a blank and a medium cultured with bacteria, without the extract, was used as a positive control.

1 $${\text{Inhibition}}( \%) = \frac{{\left( {{\text{C}} - {\text{B}}} \right) - \left( {{\text{T}} - {\text{B}}} \right)}}{{\left( {{\text{C}} - {\text{B}}} \right)}} \times 100$$

C is the average absorption of control wells, B is the average absorption of blank wells, and T is the average absorption of treated wells.

### Effect of sub-MBIC of the extract on biofilm-embedded cells

The impact of low concentrations of the extract on the cells living inside the biofilm was studied through counting colony-forming units (CFU/mL) (Rodríguez-Lázaro et al. 2018). Briefly, 200 µl of the sub-MBIC concentration of the extract (0.098 mg/ml) was poured into wells of a 96-well plate. Then, 20 µl of overnight isolated MRSA strains (10^6^ CFU/mL) was added to the wells and the plate was incubated at 37 °C  for 24 h. After the biofilm formed, the wells continent was removed and the wells were washed three times with 200 µl PBS, then 100 µl of PBS was added to each well and biofilm cells were scraped off by sterile toothpicks. The obtained suspension was then transferred to a tube and its 10-fold dilutions were prepared and cultured on Müller-Hinton agar plates. After 24 h of incubation, the colonies were counted and calculated based on Eq. 2 (Rodríguez-Lázaro et al. 2018).

2 $${{{\text{CFU}}} /{{\text{mL}} = {{\sum {{\text{CFU}}} } /{\left( {{\text{n}}_{1} - 0.1{\text{n}}_{2} } \right)}}}}{\text{d}}$$.

ΣCFU: total number of colonies from plates containing 10–150 colonies.

n1: number of plates containing 10–150 colonies.

n2: number of plates from the following dilution (containing no less than 10 colonies).

d: dilution factor corresponding to the first set of plates containing 10–150 colonies.

### Biofilm disruption and bacterial cells survival

The ability of the extract to destroy biofilm structures and kill bacterial cells was assessed using the microtiter plate according to the Khaleghi et al. method with some changes (Khaleghi et al. 2019). To determine the minimum biofilm eradication concentration (MBEC), 200 µl of the bacterial suspension of each strain (0.5 McFarland) was poured into the wells and incubated for 24 h. The wells were then drained and washed with PBS, followed by filling with 200 µl of the extract with concentrations of 0.05 to 50 mg/ml. After 24 h of incubation, the wells were drained, washed, and stained with CV. Finally, the stained biofilm was dissolved in 150 µl acetic acid and its absorbance was recorded, at 490 nm.

In order to evaluate the survival percentage of bacteria living inside the biofilm treated with the extract, the activity of the dehydrogenase enzyme of the bacterial cells was measured by 2,3,5-triphenyl tetrazolium chloride (TTC, Sigma, Germany). For this purpose, at first, the procedure mentioned above was followed. Then, instead of the CV, the cells were treated with TCC (2 %) for 2 h and the wells’ absorbance was measured at 450 nm.

### Evaluation of the biofilm genes expression

#### Total RNA extraction and cDNA synthesis

To assess the impact of the extract on the expression of the genes associated with biofilm, 10^6^ CFU/ml of the isolated strains were inoculated into the TSB medium containing the sub-MBIC concentration of *M. communis* extract (0.098 mg/mL) and incubated for 12 h at 37 °C. The medium was then centrifuged (10,000 × *g* for 20 min at 25 °C) and total RNA was extracted from the deposited cells using the NucleoSpin®RNAII kit (MACHEREY-NAGEL; Germany) according to the manufacturer’s instructions. The same MRSA strain grown in the TSB medium free of the plant extract was used as a control. Viva 2-steps RT-PCR Kit (Vivantis, Malaysia) was applied to synthesis the cDNA by using 5 µg of total RNA and 1 µl of the random hexamer. In every cDNA synthesis run, two negative controls without template and MMuLV RT were applied.

#### Real-Time PCR

Real-time RT-qPCR was performed using 10 µL 2X qPCR GreenMaster (Jena Bioscience GmbH, Germany), 2 µL cDNA, and 150 nM of each primer, mentioned in Table 2 in a 20 µL, with final volume reaction on Rotor-Gene 3000 (Corbett Research, Australia). The following temperature conditions were used in this experiment; 95 °C for 30 s, followed by 45 cycles of 95 °C for 15 s, and 66 °C for 40 s. A 72–99 °C ramp with one-degree increases every 5 s was induced to melting analysis. The data were collected on the FAM/SYBR channel, exported to an Excel worksheet, and analyzed with the LinRegPCR program (version 2015). In order to obtain the expression value, the initial concentration of every sample was normalized against 16 S reference gene concentration. The cDNA template of the untreated *S. aureus* was considered as a calibrator, and each experiment was repeated at least three times (Yaghoobi et al. 2018).

### Statistical analysis

The SPSS software version 16 was applied to statistical analysis of the obtained data through one-way ANOVA and Duncan test. The *p*-value < 0.05 was considered significant. It should be noted that all tests were performed with three replications.

## Results

### Identification of MRSA strains and screening their antibiotic resistance

Among 40 *Staphylococcus aureus* strains isolated from clinical specimens (wound, abscess, ear, and blood samples), 26 strains (65 %) were able to grow on MHA medium containing 4 % NaCl and 6 µg / mL oxacillin. It was confirmed that they all were *mecA-* positive, so they were considered MRSA and applied in further experiments.

Table 3 shows the antibiotic resistance pattern of the MRSA strains isolated from clinical samples towards 11 antibiotics. The results showed that all isolated strains were simultaneously resistant to several antibiotics so that 96.15 % of the strains were resistant to more than 2 antibiotics concurrently. Also, two strains showed resistance to more than 10 antibiotics. The highest resistance was observed against oxacillin, tetracycline, gentamicin and erythromycin, respectively. Among these, only 2 strains showed resistance to vancomycin. It was also found that 11.54 % of the strains were resistant to Mupirocin.

**Table 3:** Antibiotic resistance pattern in MRSA isolated from clinical samples. It shows the number of strains that have similar antibiotic resistance patterns

Antibiotic resistance pattern											The total number and percentage of strains have a similar pattern
VAN	CIP	ERY	TET	GEN	CRO	AMK	MUP	SXT	CHL	OX	
R	R	R	R	R	R	R	R	R	R	R	1 (3.85 %)
R	R	R	R	R	R	R	S	R	R	R	1 (3.85 %)
S	R	R	R	R	R	R	R	S	R	R	2 (7.69 %)
S	R	R	R	R	R	R	S	R	S	R	4 (15.38 %)
S	R	R	R	R	R	R	S	S	S	R	3 (11.54 %)
S	R	R	R	R	S	S	S	R	S	R	6 (23.08 %)
S	R	R	R	R	R	S	S	S	S	R	4 (15.38 %)
S	S	R	R	R	S	R	S	S	S	R	2 (7.69 %)
S	S	S	R	R	S	S	S	S	S	R	2 (7.69 %)
S	S	S	R	S	S	S	S	S	S	R	1 (3.85 %)

Antibiotics: *VAN* Vancomycin, *CIP* Ciprofloxacin, *ERY* Erythromycin, *TET* Tetracycline, *GEN* Gentamycin, *CRO* Ceftriaxone, *AMK *Amikacin, *MUP* Mupirocin, *SXT* Trimethoprim/Sulfamethoxazole, *CHL* Chloramphenicol, *OX* Oxacillin

### Antibacterial and antibiofilm activity of the extract

The results of the antibacterial effect of *M. communis* extract on 26 identified MRSA strains are shown in Table 4. According to the results, this extract showed great anti-staphylococcal activity. As the diameter of the growth inhibition zone of more than one-fourth of the strains exposed to the extract was more than 10 mm and related 50 % of them, it was between 11 and 15 mm. Also, regarding six strains (23.08 %), including Sa5, Sa8, Sa12, Sa14, Sa19, and Sa21, it was more than 15 mm. Noteworthy, the highest antibacterial activity of the plant extract was observed against Sa12 with the inhibition zone diameter (IZD) of 17.67 ± 0.29 mm. In contrast, the lowest activity was observed towards the Sa9 with the IZD of 9 ± 0.50 mm.

**Table 4:** The *M. communis* extract’s MIC, MBC, and MBIC toward MRSA strains isolated from clinical samples

Bacteria	IZD (mm)	*M. communis* extract (mg/ml)		
		MIC	MBC	MBIC
Sa1	12.50 ± 1.32	6.25	12.5	3.125
Sa2	9.83 ± 0.76	25	50	12.5
Sa3	13.33 ± 1.26	6.25	12.5	3.125
Sa4	12.17 ± 1.04	6.25	12.5	3.125
Sa5	15.67 ± 0.58	3.125	6.25	0.78
Sa6	14.67 ± 0.58	6.25	12.5	1.56
Sa7	12.17 ± 0.76	12.5	25	3.125
Sa8	16.33 ± 1.26	3.125	6.25	0.39
Sa9	9 ± 0.50	12.5	25	6.25
Sa10	10.17 ± 0.29	12.5	25	6.25
Sa11	10.00 ± 0.50	12.5	50	3.125
Sa12	17.67 ± 0.29	1.56	3.125	0.195
Sa13	13.67 ± 1.15	3.125	6.25	0.39
Sa14	17.40 ± 0.36	1.56	3.125	0.195
Sa15	13.67 ± 0.58	3.125	6.25	0.78
Sa16	9.83 ± 0.76	25	50	6.25
Sa17	10.00 ± 0.50	12.5	50	6.25
Sa18	9.83 ± 0.76	25	50	12.5
Sa19	15.17 ± 0.76	6.25	12.5	0.195
Sa20	12.17 ± 1.26	12.5	25	3.125
Sa21	17.33 ± 0.58	3.125	6.25	0.78
Sa22	14.33 ± 1.15	6.25	12.5	1.56
Sa23	12.50 ± 0.50	6.25	12.5	3.125
Sa24	12.17 ± 0.80	12.5	50	6.25
Sa25	12.33 ± 0.58	6.25	12.5	3.125
Sa26	11.50 ± 0.87	12.5	50	6.25
* *S. aureus* ATCC 33591	13.50 ± 0.50	3.125	6.25	1.56

*IZD* inhibition zone diameter, *MIC* minimum inhibitory concentration, *MBC* minimum bactericidal concentration, *MBIC* minimum biofilm-inhibitory concentration. *Standard Methicillin-Resistant *S. aureus* strain

Concerning minimum inhibitory/bactericidal concentrations of the *M. communis* extract, it was found that the range of the minimum inhibitory concentration (MIC) about the strains was between 1.56 and 25 mg/ml and the minimum bactericidal concentrations (MBC) ranged from 3.125 to 50 mg/ml (Table 4).

Moreover, Table 4 shows the minimum concentration of the extract needs to inhibit biofilm formed by each strain. In agreement with the antibacterial activity results, the lowest biofilm inhibitory concentrations were observed toward Sa5, Sa8, Sa12, Sa14, Sa19, and Sa21, indicating these are more susceptible to this extract than others. Hence, these strains were selected for subsequent studies.

### Presence of biofilm-associated genes and biofilm formation

According to the results, the *icaA* and *sarA* genes presented in 5 out of 6 strains, among them 4 strains carried both genes. Also, 4 strains had *icaD* and 2 of them had *bap* genes. Noteworthy, two strains, Sa5 and Sa12, had all four *icaA*, *icaD*, *bap* and *sarA* genes. Not surprisingly, assessing the biofilm formation capacity of these strains showed that the majority of them (66.67 %) strongly produced biofilm (Table 5).

**Table 5:** Biofilm formation capability and presence/absence of genes associated with biofilm formation in MRSA strains isolated from clinical specimens

Strains	Biofilm formation	Presence of biofilm associated-genes			
		*icaA*	*icaD*	*bap*〹	*SarA*
Sa5	+++	+	+	+	+
Sa8	+++	+	+	–	–
Sa12	+++	+	+	+	+
Sa14	+++	+	+	−	+
Sa19	+	–	–	–	+
Sa21	++	+	–	–	+
+++: strong biofilm formation (OD492 > 1.2), ++: medium-positive biofilm formation (1.2˃ OD492 > 0.6) and +: weak biofilm formation (0.6 ˃OD492 > 0.3).					

### The antibiofilm property of the ***M. communis*** extract

**Fig. 1:**
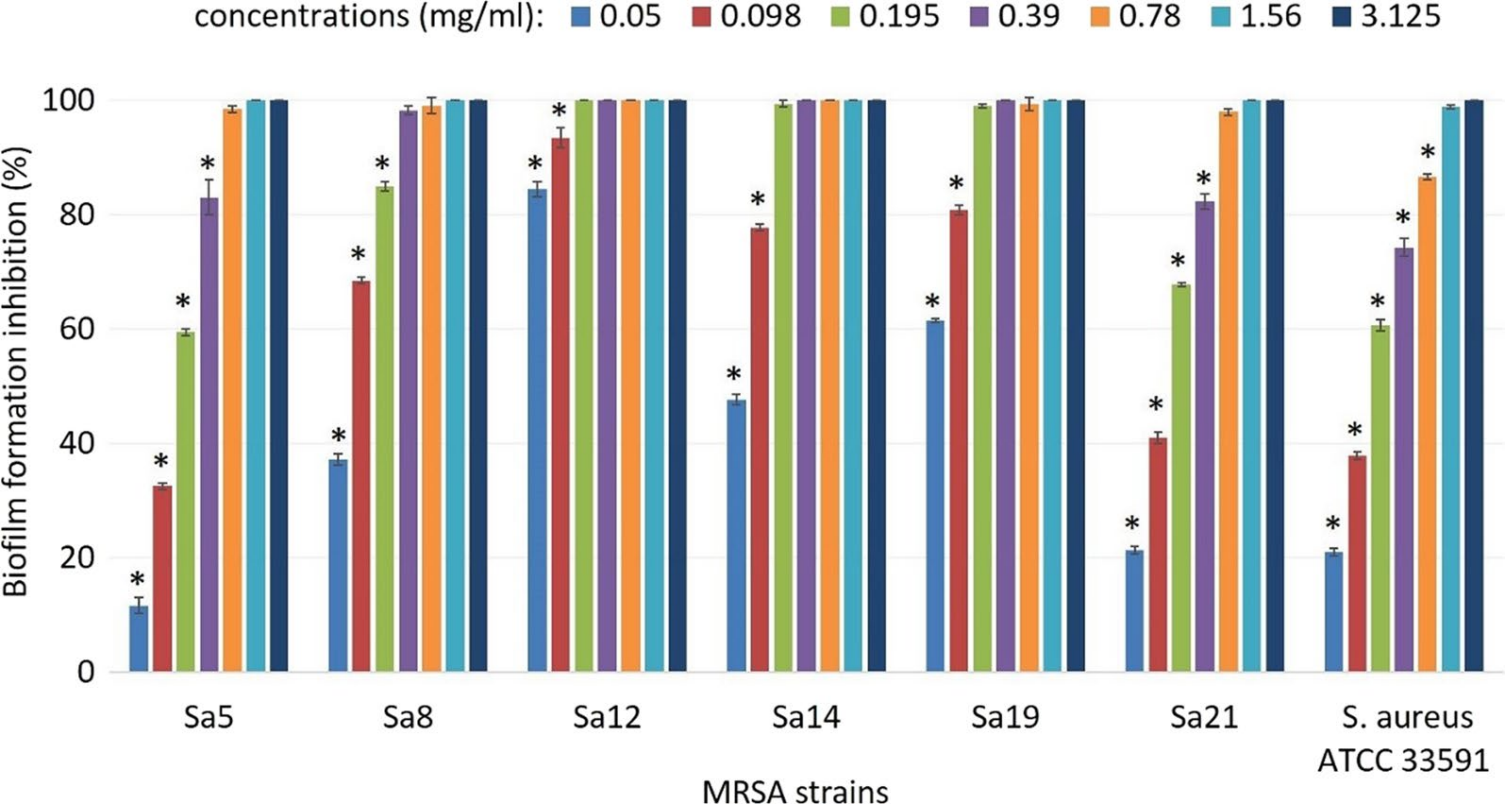
The presence of the biofilm formation inhibition regard to strains Sa5, Sa8, Sa12, Sa14, Sa19, Sa21 and *S. aureus* ATCC 33591. Error bars represent standard deviations (SD). *P < 0.05

To determine the maximum antibiofilm efficiency of the plant extract, its impact on the biofilm formation of the isolates shown the most susceptibility toward the extract was evaluated. According to the results, the sub-MIC concentration of the extract inhibited the biofilm formation in all 6 strains by more than 97 %, in a dose-dependent manner (Fig. 1). Among all strains, the Sa12 was affected much more than others, so that when it was exposed to the concentrations ≥ 0.195 mg/ml its biofilm formation was entirely inhibited. Also, lower concentrations could stop this process by more than 85 %.

### Effect of sub-MBIC of the extract on biofilm structure and living cells

**Fig. 2:**
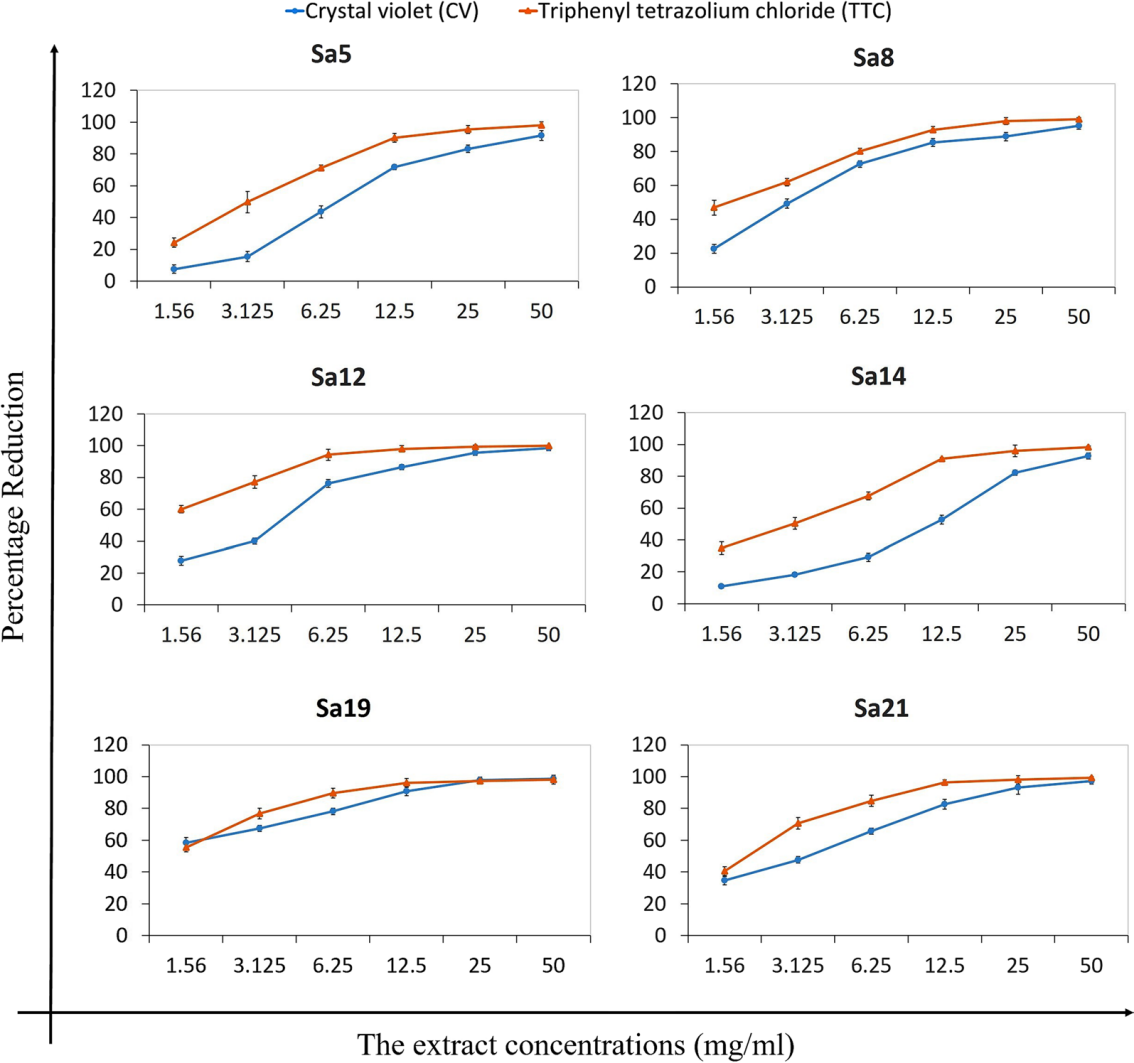
The percentage of reduction of viable bacterial cells (log10 CFU/mL) after exposure to sub-MIC concentration (0.098 mg/ml) of the *M. communis* extract

Figure 2 shows the percentage of biofilm destruction and elimination of the cells settled in biofilm determined through CV and TTC staining, respectively. According to the results, the extract was able not only to destroy the biofilm formed already but also to kill the bacterial cells established in the biofilm, in a concentration-dependent manner. The concentrations of 25 and 50 mg/ml of the ethanolic extract destroyed more than 80 % of the biofilm formed by all strains. Furthermore, the results of TTC staining showed that 12.5 mg/ml of the extract killed more than 90 % of the cells inhabited in the biofilm.

Given that the biofilm of the Sa12 was affected by the *M. communis* extract more than others, as well as due to the presence of all genes involved in biofilm formation, the effect of sub-MBIC concentration on the expression of these genes was investigated just related to this strain.

### Expression of genes encoding adhesion factors and biofilm

**Fig. 3:**
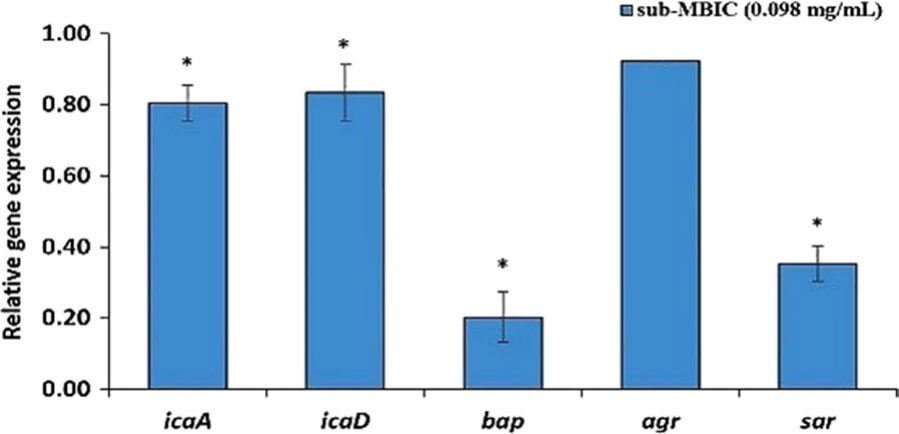
Effect of sub-MBIC concentration of the extract of *M. communis* on the expression of *icaA*, *icaD*, *bap*, *sar*, and *agr* genes in Sa12 strain. Gene expression data were normalized to the 16 S reference

Figure 3 shows how the sub-MBIC concentration of the *M. communis* extract affected the expression of genes involved in biofilm formation in the Sa12 strain. According to the results, this extract significantly decreased the expression of *icaA*, *icaD*, *bap*, and *sarA* genes compared to the control (p < 0.05). Also, it imposed the most effect on the expression of the *bap* so that in the treated cells the expression of this gene reduced about 5 times compared to the control. Also, the expression of *sarA*, *icaA* and *icaD* decreased about 3-fold, 2-fold and 1.4-fold, respectively. Although the sub-MBIC concentration of the extract diminished the expression of *agr*, no substantial difference was observed compared to the control group, based on the statistical analyses.

## Discussion

Antibiotics are mass-produced at an estimated scale of about 100,000 tons per year worldwide, and their use remarkably affects the lives of bacteria living on earth. More strains of pathogens have become resistant to antibiotics, and some have developed resistance to various antibiotics and chemotherapeutic agents, emerging multidrug-resistant bacteria. Indeed, some strains have shown resistance to almost all regularly available agents. The methicillin-resistant *Staphylococcus aureus* (MRSA) is a notorious case. This strain is resistant to methicillin, which was prescribed to fight against penicillinase-producing *S. aureus*. MRSA is usually resistant to aminoglycosides, macrolides, tetracycline, chloramphenicol, and lincosamides, too (Nikaido 2009).

As our results revealed, a considerable proportion of the isolated strains showed resistance to the antibiotics. Additionally, the presence of the *mecA* gene was confirmed in the strains. This gene is a part of a staphylococcal chromosome cassette mec (SCCmec), which is a mobile genetic element that may also contain genetic structures that encodes resistance to non-β-lactam antibiotics (Wielders et al. 2002).

Thanks to genetic mutations and gene transfer mechanisms, pathogens are becoming more and more resistant; subsequently we have to annually phase out some conventional antibiotics because they gradually become inefficient. Therefore, the discovery of effective antibacterial agents is of extreme urgency.

According to evidence from the civilizations of China, India and the Near East, the use of some plants as remedies dates back to thousands of years ago. These plants are still used in traditional medicine around the world (Kalaivani et al. 2012). Plants have developed their defense mechanisms through the co-evolution with pathogenic bacteria and the production of secondary metabolites against parasites (Datta and Nandy, 2011). The antimicrobial and anti-biofilm properties of some plant extracts have been proven by numerous studies. This activity is generally attributed to bioactive components such as terpenoids, saponins, alkaloids, and glycosides in plant extracts (Mathlouthi et al. 2021; Ozma et al. 2021; Thakur et al. 2020).

As the GC-MS analysis indicated, the *M. communis* extract consists of several bioactive compounds, and 2-Furancarboxaldehyde, 5-(hydroxymethyl) derived from furaldehyde with the furan base is the most abundant compound in the extract. It has already been demonstrated that furan derivatives in certain plants function as antibacterial and antibiofilm agents (Sethupathy et al. 2015; Subramenium et al. 2018). In this regard, the antibacterial activity of the ethanolic leaf extract of *M. communis* originated from the southwestern region of Saudi Arabia, Jazan, have recently been reported by researchers (Mir et al. 2020). The extract inhibited the growth of several gram-positive bacteria and acid-fast *M. smegmatis*, while it did not show a considerable activity against gram-negative strains. The researchers argued that the secondary metabolites of the ethanolic extract of *M. communis* might target the peptidoglycan layer and penetration and accumulation of compounds in the cell membrane could not be suggested as a central mechanism of action. Furthermore, an aqueous extract of *M. communis* has been reported to be applied as an eco-friendly, economic and biological agent to synthesis silver nanoparticles. The biologically synthesized nanoparticles were active against both gram-positive and gram-negative bacteria. Such promising results could be related to both silver nanoparticles and the extract components that cover them (Alyousef et al. 2019; Khorrami et al. 2020).

The antibiofilm activity of the *M. communis* extract and its essential oil towards various bacterial strains has been previously investigated by scientists attributing this activity to the bioactive components of the extract (Cannas et al. 2014; Feuillolay et al. 2016; Saviuc et al. 2015). Yet, no reliable study on the effects of the extract on the MRSA biofilm formation is available. To achieve more details, we investigated how the biofilm produced by the strains, as well as the bacterial cells inside the structure, is affected by the extract.

Recent progress in clarifying the role of the genes involved in providing the substrate of biofilm formation in staphylococcal biofilm development has raised our insight into the pathogenesis of these strains. Production of the polysaccharide intercellular adhesion (PIA) or polymeric N-acetyl-glucosamine (PNAG), an extracellular polysaccharide adhesion, by enzymes encoded via ica operon, is currently the most known biofilm mechanism in staphylococci (O’Gara 2007). The *icaA* gene products a transmembrane protein with homology to N-acetylglucosaminyl transferases, which requires the product of the *icaD* gene to function optimally (O’Gara 2007). According to the results of the present study, the *M. communis* significantly down-regulated the expression of both *icaA* and *icaD* genes. Also, it significantly reduced the expression of the *sarA* and *bap* genes in the Sa12 strain. It has been reported that the *sarA* locus is required for ica operon transcription, PIA/PNAG production, and biofilm formation in *S. aureus* (Beenken et al. 2003; Valle et al. 2003). Furthermore, the biofilm-associated protein (Bap) is needed for initial adherence and intercellular accumulation during biofilm progress. *SarA* acts as a transcriptional initiator of the *bap* gene, too; therefore, it positively regulates Bap-mediated biofilm development (Trotonda et al. 2005). Consequently, it could be argued that the *M. communis* inhibit biofilm formation by disrupting the expression of essential genes, as the primary mechanism.

Concerning the global regulator *agr*, the extract did not show a significant effect on the expression of this gene. It has already been revealed that mutation of *agr* has a neutral effect on the biofilm formation process (Beenken et al. 2003) or can lead to increased biofilm formation (Valliammai et al. 2019). Moreover, the influence that the *agr* gene’s changes impose on biofilm formation can be positive, neutral, or negative, depending on growth conditions. Perhaps it reflects the sensitivity of the Agr system to external environmental signals.

**Fig. 4:**
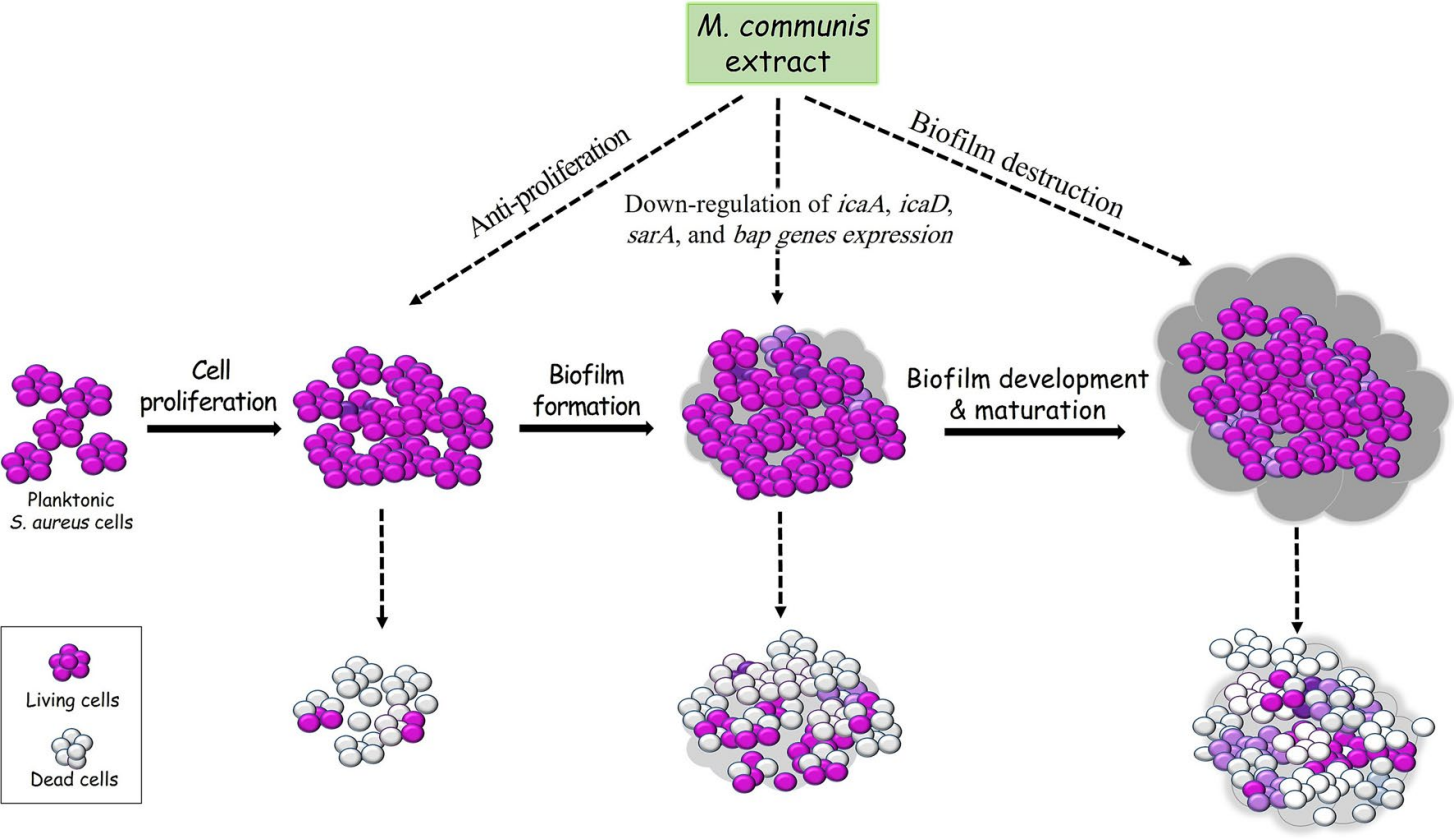
Illustrates the biofilm formation and development process, and the ways through which the extract can combat MRSA. *M. communis* extract directly inhibits cells proliferation and prevents biofilm formation through down-regulation of genes encoding adhesion factors and biofilm substances. It also ruins the established biofilm and kills the cells living inside the biofilm

Concluding, the prevalence of MRSA in *S. aureus* isolated from clinical samples is worrying. Yet, the ethanolic extract of *M. communis* showed great potential as an effective antibacterial agent to combat MRSA strains. As illustrated in Fig. 4, the extract not only inhibits bacterial cell proliferation and kills them, but also can inhibit the biofilm formation, destroys the pre-formed biofilm, and eradicates the bacterial cells settled inside the biofilm through down-regulation of the biofilm-associated genes (*icaA*, *icaD*, *sarA* and *bap*). These features indicate the extract a reliable antibiofilm agent that nips the biofilm formation in the bud.

## Data Availability

Not applicable.
